# (5′′*E*)-1′′-Benzyl-1′-methyl-5′′-[4-(methyl­sulfan­yl)benzyl­idene]-4′-[4-(methyl­sulfan­yl)phen­yl]dispiro­[indoline-3,2′-pyrrolidine-3′,3′′-piperidine]-2,4′′-dione dichloro­methane solvate

**DOI:** 10.1107/S1600536809054774

**Published:** 2009-12-24

**Authors:** Yongjiang Hou

**Affiliations:** a School of Environmental Science and Engineering, Hebei University of Science and Technology. 050018 Shijiazhuang, Hebei province, People’s Republic of China

## Abstract

In the title compound, C_38_H_36_N_3_O_2_S_2_·CH_2_Cl_2_, the 2-oxindole ring is almost planar (r.m.s. deviation = 0.032 Å), the pyrrolidine ring adopts a twist conformation and the piperidone ring exists as a chair. Three short C—H⋯O intra­molecular contacts occur. In the crystal, mol­ecules are linked by C—H⋯O and C—H⋯N inter­actions. The dichloro­methane solvent mol­ecule is disordered over two orientations in a 0.765 (11):0.235 (11) ratio.

## Related literature

For background to dispiro ring systems, see: Kobayashi *et al.* (1991 ▶).
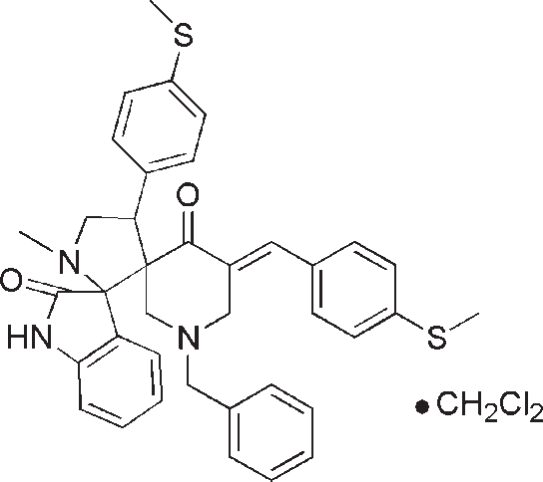


## Experimental

### 

#### Crystal data

C_38_H_36_N_3_O_2_S_2_·CH_2_Cl_2_
*M* *_r_* = 715.74
Monoclinic, 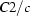

*a* = 36.456 (7) Å
*b* = 8.6164 (17) Å
*c* = 26.184 (5) Å
β = 119.58 (3)°
*V* = 7153 (2) Å^3^
*Z* = 8
Mo *K*α radiation
μ = 0.34 mm^−1^
*T* = 113 K
0.20 × 0.18 × 0.12 mm

#### Data collection

Rigaku Saturn CCD diffractometer
Absorption correction: multi-scan (*CrystalClear*; Rigaku, 2005 ▶) *T*_min_ = 0.936, *T*_max_ = 0.961
20568 measured reflections
6263 independent reflections
2890 reflections with *I* > 2σ(*I*)
*R*_int_ = 0.116

#### Refinement

*R*[*F* ^2^ > 2σ(*F* ^2^)] = 0.059
*wR*(*F* ^2^) = 0.154
*S* = 1.01
6263 reflections
455 parameters
40 restraints
H-atom parameters constrained
Δρ_max_ = 0.44 e Å^−3^
Δρ_min_ = −0.46 e Å^−3^

### 

Data collection: *CrystalClear* (Rigaku, 2005 ▶); cell refinement: *CrystalClear*; data reduction: *CrystalClear*; program(s) used to solve structure: *SHELXS97* (Sheldrick, 2008 ▶); program(s) used to refine structure: *SHELXL97* (Sheldrick, 2008 ▶); molecular graphics: *SHELXTL* (Sheldrick, 2008 ▶); software used to prepare material for publication: *SHELXTL*.

## Supplementary Material

Crystal structure: contains datablocks I, global. DOI: 10.1107/S1600536809054774/hb5259sup1.cif

Structure factors: contains datablocks I. DOI: 10.1107/S1600536809054774/hb5259Isup2.hkl

Additional supplementary materials:  crystallographic information; 3D view; checkCIF report

## Figures and Tables

**Table 1:** Hydrogen-bond geometry (Å, °)

*D*—H⋯*A*	*D*—H	H⋯*A*	*D*⋯*A*	*D*—H⋯*A*
C1—H1*A*⋯O2	0.97	2.53	3.149 (5)	122
C7—H7*B*⋯O2	0.97	2.37	2.971 (5)	119
C10—H10⋯O1	0.93	2.57	3.220 (5)	127
C39—H39*A*⋯N3	0.97	2.44	3.405 (10)	175
C39—H39*B*⋯O1	0.97	2.53	3.140 (7)	121

**Fig. 1.**
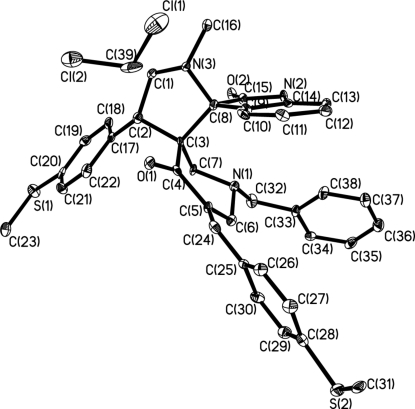
The molecular structure of (I), drawn with 30% probability ellipsoids

## supplementary crystallographic information

### Comment

In this paper, the structure of the title compound, (I), is reported. The molecular structure of (I) is illustrated in Fig. 1. There exists a dispiro rings in the molecule which was consist of a 2-oxindole ring, a pyrrolidine ring and a piperidone rings. The pyrrolidine ring is not planar, with twist conformations. 2-oxindole ring (C15/N2/C14/C13/C12/C11/C10/C9/C8) is nearly planar that the mean deviation from this plane is 0.0148 (3)%A.

The piperidone ring has the usual chair conformation. There exist intermolecular O—H—N hydrogen bonds.

### Experimental

A mixture of 1-benzyl-3,5-bis-(4-methylsulfanyl-benzylidene)-piperidin-4-one (2 mmol), isatin (2 mmol) and sarcosine (2 mmol) were refluxed in methanol (80 ml) until the disapperence of the starting material as evidenced by the TLC. After the reaction was over the solvent was removed *in vacuo* and the residue was separated by column chromatography (silica gel, petroleum ether/ethyl acetate = 5:1) to give the title compound. 20 mg of the title compound was dissolved in 15 ml dichloromethane; the solution was kept at room temperature for 15 d by natural evaporation to give colourless blocks of (I).

### Refinement

All H atoms were fixed geometrically and treated as riding with C—H = 0.96Å (methyl), 0.97Å (methylene), 0.98Å (methine) with *U*_iso_(H) = 1.2*U*_eq_(C) or *U*_iso_(H)= 1.5*U*_eq_(methine).

### Crystal data

**Table:**

C_38_H_36_N_3_O_2_S_2_·CH_2_Cl_2_	*F*(000) = 3000
*M**_r_* = 715.74	*D*_x_ = 1.329 Mg m^−^^3^
Monoclinic, *C*2/*c*	Melting point: 473 K
Hall symbol: -C 2yc	Mo *K*α radiation, λ = 0.71073 Å
*a* = 36.456 (7) Å	Cell parameters from 8719 reflections
*b* = 8.6164 (17) Å	θ = 1.6–27.9°
*c* = 26.184 (5) Å	µ = 0.34 mm^−^^1^
β = 119.58 (3)°	*T* = 113 K
*V* = 7153 (2) Å^3^	Block, colourless
*Z* = 8	0.20 × 0.18 × 0.12 mm

### Data collection

**Table:**

Rigaku Saturn CCD diffractometer	6263 independent reflections
Radiation source: rotating anode	2890 reflections with *I* > 2σ(*I*)
confocal	*R*_int_ = 0.116
Detector resolution: 7.31 pixels mm^-1^	θ_max_ = 25.0°, θ_min_ = 2.3°
ω and φ scans	*h* = −36→43
Absorption correction: multi-scan (*CrystalClear*; Rigaku, 2005)	*k* = −10→10
*T*_min_ = 0.936, *T*_max_ = 0.961	*l* = −31→30
20568 measured reflections	

### Refinement

**Table:**

Refinement on *F*^2^	Primary atom site location: structure-invariant direct methods
Least-squares matrix: full	Secondary atom site location: difference Fourier map
*R*[*F*^2^ > 2σ(*F*^2^)] = 0.059	Hydrogen site location: inferred from neighbouring sites
*wR*(*F*^2^) = 0.154	H-atom parameters constrained
*S* = 1.01	*w* = 1/[σ^2^(*F*_o_^2^) + (0.028*P*)^2^] where *P* = (*F*_o_^2^ + 2*F*_c_^2^)/3
6263 reflections	(Δ/σ)_max_ = 0.001
455 parameters	Δρ_max_ = 0.44 e Å^−^^3^
40 restraints	Δρ_min_ = −0.46 e Å^−^^3^

### Special details

**Table:**

Geometry. All e.s.d.'s (except the e.s.d. in the dihedral angle between two l.s. planes) are estimated using the full covariance matrix. The cell e.s.d.'s are taken into account individually in the estimation of e.s.d.'s in distances, angles and torsion angles; correlations between e.s.d.'s in cell parameters are only used when they are defined by crystal symmetry. An approximate (isotropic) treatment of cell e.s.d.'s is used for estimating e.s.d.'s involving l.s. planes.
Refinement. Refinement of *F*^2^ against ALL reflections. The weighted *R*-factor *wR* and goodness of fit *S* are based on *F*^2^, conventional *R*-factors *R* are based on *F*, with *F* set to zero for negative *F*^2^. The threshold expression of *F*^2^ > σ(*F*^2^) is used only for calculating *R*-factors(gt) *etc*. and is not relevant to the choice of reflections for refinement. *R*-factors based on *F*^2^ are statistically about twice as large as those based on *F*, and *R*- factors based on ALL data will be even larger.

### Fractional atomic coordinates and isotropic or equivalent isotropic displacement parameters (Å^2^)

**Table:**

	*x*	*y*	*z*	*U*_iso_*/*U*_eq_	Occ. (<1)
S1	0.81245 (4)	−0.42806 (12)	0.32967 (5)	0.0300 (3)	
S2	1.03858 (4)	−0.26067 (12)	0.01917 (5)	0.0282 (3)	
O1	0.93859 (8)	0.1218 (3)	0.23595 (11)	0.0217 (7)	
O2	0.77000 (8)	0.1837 (3)	0.07939 (11)	0.0206 (6)	
N1	0.84168 (10)	−0.0525 (3)	0.08359 (13)	0.0167 (7)	
N2	0.80248 (10)	0.2974 (3)	0.03269 (13)	0.0161 (8)	
N3	0.84549 (10)	0.3613 (3)	0.17922 (13)	0.0162 (7)	
C1	0.83077 (13)	0.2676 (4)	0.21221 (16)	0.0197 (9)	
H1A	0.8014	0.2384	0.1879	0.024*	
H1B	0.8342	0.3225	0.2466	0.024*	
C2	0.85950 (12)	0.1269 (4)	0.22927 (17)	0.0188 (9)	
H2	0.8873	0.1605	0.2604	0.023*	
C3	0.86374 (12)	0.0969 (4)	0.17302 (15)	0.0123 (8)	
C4	0.90973 (12)	0.0694 (4)	0.18967 (16)	0.0161 (9)	
C5	0.91820 (12)	−0.0144 (4)	0.14716 (16)	0.0148 (9)	
C6	0.88378 (11)	−0.1081 (4)	0.09815 (16)	0.0178 (9)	
H6A	0.8870	−0.1016	0.0636	0.021*	
H6B	0.8866	−0.2162	0.1099	0.021*	
C7	0.83915 (12)	−0.0466 (4)	0.13777 (15)	0.0163 (9)	
H7A	0.8512	−0.1400	0.1607	0.020*	
H7B	0.8099	−0.0395	0.1284	0.020*	
C8	0.84714 (12)	0.2525 (4)	0.13602 (16)	0.0154 (9)	
C9	0.87097 (11)	0.3202 (4)	0.10800 (16)	0.0151 (9)	
C10	0.91286 (12)	0.3682 (4)	0.13121 (17)	0.0194 (9)	
H10	0.9318	0.3559	0.1711	0.023*	
C11	0.92606 (12)	0.4327 (4)	0.09561 (17)	0.0211 (10)	
H11	0.9539	0.4651	0.1116	0.025*	
C12	0.89836 (13)	0.4514 (4)	0.03508 (18)	0.0246 (10)	
H12	0.9081	0.4924	0.0111	0.029*	
C13	0.85601 (12)	0.4080 (4)	0.01113 (17)	0.0177 (9)	
H13	0.8370	0.4218	−0.0286	0.021*	
C14	0.84364 (12)	0.3449 (4)	0.04796 (16)	0.0163 (9)	
C15	0.80162 (12)	0.2343 (4)	0.08046 (16)	0.0192 (9)	
C16	0.82163 (14)	0.5041 (4)	0.15488 (17)	0.0275 (11)	
H16A	0.7928	0.4790	0.1275	0.041*	
H16B	0.8336	0.5608	0.1350	0.041*	
H16C	0.8228	0.5664	0.1861	0.041*	
C17	0.84726 (12)	−0.0104 (4)	0.25245 (15)	0.0145 (9)	
C18	0.80632 (12)	−0.0352 (5)	0.24449 (16)	0.0223 (10)	
H18	0.7851	0.0364	0.2230	0.027*	
C19	0.79734 (13)	−0.1614 (5)	0.26757 (17)	0.0234 (10)	
H19	0.7700	−0.1745	0.2607	0.028*	
C20	0.82776 (13)	−0.2718 (4)	0.30111 (16)	0.0203 (10)	
C21	0.86876 (13)	−0.2481 (4)	0.31048 (17)	0.0233 (10)	
H21	0.8900	−0.3187	0.3329	0.028*	
C22	0.87787 (13)	−0.1194 (5)	0.28639 (17)	0.0247 (10)	
H22	0.9052	−0.1060	0.2932	0.030*	
C23	0.86044 (12)	−0.5343 (5)	0.37319 (17)	0.0286 (11)	
H23A	0.8693	−0.5819	0.3479	0.043*	
H23B	0.8557	−0.6130	0.3952	0.043*	
H23C	0.8820	−0.4646	0.3997	0.043*	
C24	0.95764 (12)	0.0014 (4)	0.15326 (17)	0.0207 (10)	
H24	0.9759	0.0637	0.1846	0.025*	
C25	0.97545 (12)	−0.0613 (4)	0.11979 (16)	0.0165 (9)	
C26	1.00906 (12)	0.0163 (4)	0.11886 (17)	0.0233 (10)	
H26	1.0188	0.1081	0.1400	0.028*	
C27	1.02810 (12)	−0.0368 (5)	0.08835 (18)	0.0254 (10)	
H27	1.0496	0.0210	0.0883	0.031*	
C28	1.01570 (12)	−0.1768 (4)	0.05726 (17)	0.0226 (10)	
C29	0.98184 (13)	−0.2554 (4)	0.05714 (18)	0.0249 (10)	
H29	0.9723	−0.3472	0.0359	0.030*	
C30	0.96232 (13)	−0.2024 (4)	0.08701 (18)	0.0232 (10)	
H30	0.9402	−0.2590	0.0859	0.028*	
C31	1.07740 (13)	−0.1205 (4)	0.02636 (19)	0.0291 (11)	
H31A	1.0637	−0.0238	0.0093	0.044*	
H31B	1.0918	−0.1576	0.0064	0.044*	
H31C	1.0974	−0.1052	0.0672	0.044*	
C32	0.80877 (13)	−0.1550 (4)	0.03998 (16)	0.0247 (10)	
H32A	0.7820	−0.1289	0.0374	0.030*	
H32B	0.8154	−0.2614	0.0537	0.030*	
C33	0.80392 (12)	−0.1451 (4)	−0.02036 (16)	0.0191 (9)	
C34	0.78570 (11)	−0.2681 (4)	−0.05875 (17)	0.0180 (9)	
H34	0.7792	−0.3589	−0.0457	0.022*	
C35	0.77693 (13)	−0.2570 (5)	−0.11688 (18)	0.0288 (11)	
H35	0.7646	−0.3402	−0.1423	0.035*	
C36	0.78647 (13)	−0.1237 (5)	−0.13671 (18)	0.0306 (11)	
H36	0.7800	−0.1160	−0.1757	0.037*	
C37	0.80560 (13)	−0.0015 (5)	−0.09880 (18)	0.0288 (11)	
H37	0.8126	0.0880	−0.1119	0.035*	
C38	0.81437 (13)	−0.0121 (5)	−0.04127 (17)	0.0244 (10)	
H38	0.8275	0.0706	−0.0159	0.029*	
Cl1	0.95562 (15)	0.6560 (5)	0.26917 (14)	0.0621 (10)	0.765 (11)
C39	0.9453 (3)	0.4623 (7)	0.2825 (3)	0.050 (2)	0.765 (11)
H39A	0.9163	0.4362	0.2546	0.060*	0.765 (11)
H39B	0.9634	0.3917	0.2762	0.060*	0.765 (11)
Cl1'	0.9728 (4)	0.5901 (17)	0.2710 (4)	0.060 (3)	0.235 (11)
C39'	0.9325 (4)	0.508 (4)	0.2821 (6)	0.049 (5)	0.235 (11)
H39C	0.9115	0.5868	0.2750	0.058*	0.235 (11)
H39D	0.9188	0.4246	0.2543	0.058*	0.235 (11)
Cl2	0.95372 (4)	0.43615 (14)	0.35449 (6)	0.0482 (4)	

### Atomic displacement parameters (Å^2^)

**Table:**

	*U*^11^	*U*^22^	*U*^33^	*U*^12^	*U*^13^	*U*^23^
S1	0.0261 (7)	0.0307 (6)	0.0351 (7)	−0.0029 (6)	0.0166 (6)	0.0082 (6)
S2	0.0301 (7)	0.0283 (6)	0.0334 (7)	0.0046 (5)	0.0213 (6)	0.0022 (6)
O1	0.0125 (15)	0.0322 (16)	0.0118 (15)	0.0005 (13)	−0.0006 (13)	−0.0042 (13)
O2	0.0099 (15)	0.0301 (15)	0.0189 (16)	−0.0067 (13)	0.0049 (13)	0.0066 (13)
N1	0.0142 (18)	0.0185 (16)	0.0123 (18)	−0.0033 (15)	0.0027 (15)	−0.0038 (15)
N2	0.0154 (18)	0.0216 (17)	0.0151 (18)	−0.0049 (15)	0.0104 (15)	0.0053 (15)
N3	0.0218 (19)	0.0154 (16)	0.0154 (18)	0.0015 (15)	0.0122 (16)	0.0002 (15)
C1	0.028 (3)	0.018 (2)	0.017 (2)	0.0012 (19)	0.013 (2)	−0.0018 (18)
C2	0.015 (2)	0.024 (2)	0.015 (2)	−0.0008 (19)	0.0060 (19)	−0.0012 (19)
C3	0.014 (2)	0.0155 (19)	0.009 (2)	0.0007 (18)	0.0065 (17)	0.0044 (17)
C4	0.018 (2)	0.0125 (19)	0.015 (2)	0.0009 (18)	0.007 (2)	0.0051 (18)
C5	0.012 (2)	0.022 (2)	0.013 (2)	−0.0010 (18)	0.0078 (18)	0.0014 (18)
C6	0.021 (2)	0.017 (2)	0.019 (2)	−0.0050 (19)	0.012 (2)	−0.0017 (18)
C7	0.017 (2)	0.025 (2)	0.010 (2)	0.0001 (19)	0.0094 (18)	0.0006 (18)
C8	0.013 (2)	0.022 (2)	0.011 (2)	0.0007 (19)	0.0057 (18)	−0.0002 (18)
C9	0.013 (2)	0.0135 (19)	0.017 (2)	0.0002 (18)	0.0057 (19)	0.0039 (18)
C10	0.016 (2)	0.013 (2)	0.019 (2)	0.0025 (18)	0.0008 (19)	0.0029 (18)
C11	0.013 (2)	0.019 (2)	0.030 (3)	−0.0009 (19)	0.009 (2)	0.002 (2)
C12	0.029 (3)	0.019 (2)	0.029 (3)	0.003 (2)	0.017 (2)	0.002 (2)
C13	0.018 (2)	0.021 (2)	0.015 (2)	0.0021 (19)	0.0090 (19)	0.0025 (18)
C14	0.015 (2)	0.016 (2)	0.013 (2)	0.0060 (18)	0.0032 (18)	−0.0002 (18)
C15	0.014 (2)	0.026 (2)	0.011 (2)	0.004 (2)	0.0020 (18)	0.0010 (19)
C16	0.041 (3)	0.028 (2)	0.022 (3)	0.008 (2)	0.022 (2)	0.006 (2)
C17	0.016 (2)	0.0167 (19)	0.008 (2)	−0.0033 (18)	0.0042 (18)	−0.0037 (17)
C18	0.019 (2)	0.032 (2)	0.011 (2)	0.002 (2)	0.0042 (19)	−0.001 (2)
C19	0.017 (2)	0.029 (2)	0.023 (3)	−0.001 (2)	0.010 (2)	−0.008 (2)
C20	0.025 (2)	0.029 (2)	0.011 (2)	−0.007 (2)	0.0120 (19)	−0.0068 (19)
C21	0.018 (2)	0.026 (2)	0.024 (2)	0.004 (2)	0.009 (2)	0.006 (2)
C22	0.016 (2)	0.038 (3)	0.017 (2)	−0.006 (2)	0.006 (2)	−0.003 (2)
C23	0.031 (3)	0.036 (3)	0.024 (3)	−0.007 (2)	0.018 (2)	0.003 (2)
C24	0.017 (2)	0.017 (2)	0.017 (2)	−0.0038 (19)	−0.0003 (19)	0.0012 (19)
C25	0.015 (2)	0.018 (2)	0.016 (2)	0.0048 (18)	0.0077 (19)	0.0028 (18)
C26	0.018 (2)	0.024 (2)	0.023 (2)	−0.001 (2)	0.006 (2)	−0.004 (2)
C27	0.014 (2)	0.029 (2)	0.029 (3)	−0.002 (2)	0.007 (2)	−0.002 (2)
C28	0.019 (2)	0.023 (2)	0.022 (2)	0.002 (2)	0.008 (2)	0.007 (2)
C29	0.024 (3)	0.022 (2)	0.027 (3)	0.000 (2)	0.011 (2)	−0.001 (2)
C30	0.019 (2)	0.017 (2)	0.033 (3)	−0.0006 (19)	0.013 (2)	0.002 (2)
C31	0.038 (3)	0.027 (2)	0.042 (3)	0.007 (2)	0.035 (3)	0.005 (2)
C32	0.020 (2)	0.029 (2)	0.019 (2)	−0.011 (2)	0.004 (2)	−0.004 (2)
C33	0.014 (2)	0.030 (2)	0.012 (2)	−0.001 (2)	0.0049 (19)	0.000 (2)
C34	0.014 (2)	0.017 (2)	0.024 (2)	−0.0007 (18)	0.0102 (19)	−0.0014 (19)
C35	0.025 (3)	0.037 (3)	0.020 (2)	0.000 (2)	0.008 (2)	−0.011 (2)
C36	0.034 (3)	0.033 (3)	0.019 (3)	−0.001 (2)	0.009 (2)	−0.002 (2)
C37	0.030 (3)	0.034 (2)	0.025 (3)	−0.002 (2)	0.015 (2)	0.005 (2)
C38	0.027 (2)	0.028 (2)	0.014 (2)	−0.009 (2)	0.006 (2)	−0.009 (2)
Cl1	0.062 (2)	0.0615 (18)	0.0748 (16)	−0.0259 (16)	0.0424 (15)	−0.0276 (14)
C39	0.034 (5)	0.037 (4)	0.050 (4)	0.012 (3)	−0.001 (3)	−0.027 (3)
Cl1'	0.054 (5)	0.069 (6)	0.040 (4)	−0.019 (4)	0.010 (3)	−0.005 (4)
C39'	0.040 (9)	0.053 (9)	0.047 (8)	0.001 (8)	0.017 (7)	−0.017 (8)
Cl2	0.0323 (7)	0.0441 (7)	0.0626 (9)	0.0007 (6)	0.0191 (7)	−0.0107 (7)

### Geometric parameters (Å, °)

**Table:**

S1—C20	1.760 (4)	C18—H18	0.9300
S1—C23	1.797 (4)	C19—C20	1.394 (5)
S2—C28	1.742 (4)	C19—H19	0.9300
S2—C31	1.799 (4)	C20—C21	1.405 (5)
O1—C4	1.233 (4)	C21—C22	1.394 (5)
O2—C15	1.220 (4)	C21—H21	0.9300
N1—C6	1.467 (4)	C22—H22	0.9300
N1—C7	1.467 (4)	C23—H23A	0.9600
N1—C32	1.474 (4)	C23—H23B	0.9600
N2—C15	1.378 (4)	C23—H23C	0.9600
N2—C14	1.409 (4)	C24—C25	1.429 (5)
N3—C16	1.459 (4)	C24—H24	0.9300
N3—C1	1.464 (4)	C25—C26	1.406 (5)
N3—C8	1.493 (4)	C25—C30	1.428 (5)
C1—C2	1.518 (5)	C26—C27	1.370 (5)
C1—H1A	0.9700	C26—H26	0.9300
C1—H1B	0.9700	C27—C28	1.399 (5)
C2—C17	1.495 (5)	C27—H27	0.9300
C2—C3	1.576 (5)	C28—C29	1.406 (5)
C2—H2	0.9800	C29—C30	1.370 (5)
C3—C4	1.528 (5)	C29—H29	0.9300
C3—C7	1.539 (5)	C30—H30	0.9300
C3—C8	1.588 (5)	C31—H31A	0.9600
C4—C5	1.482 (5)	C31—H31B	0.9600
C5—C24	1.373 (5)	C31—H31C	0.9600
C5—C6	1.511 (5)	C32—C33	1.503 (5)
C6—H6A	0.9700	C32—H32A	0.9700
C6—H6B	0.9700	C32—H32B	0.9700
C7—H7A	0.9700	C33—C34	1.384 (5)
C7—H7B	0.9700	C33—C38	1.402 (5)
C8—C9	1.504 (5)	C34—C35	1.395 (5)
C8—C15	1.585 (5)	C34—H34	0.9300
C9—C10	1.399 (5)	C35—C36	1.374 (5)
C9—C14	1.401 (5)	C35—H35	0.9300
C10—C11	1.361 (5)	C36—C37	1.377 (5)
C10—H10	0.9300	C36—H36	0.9300
C11—C12	1.406 (5)	C37—C38	1.380 (5)
C11—H11	0.9300	C37—H37	0.9300
C12—C13	1.400 (5)	C38—H38	0.9300
C12—H12	0.9300	Cl1—C39	1.783 (6)
C13—C14	1.363 (5)	C39—Cl2	1.767 (6)
C13—H13	0.9300	C39—H39A	0.9700
C16—H16A	0.9600	C39—H39B	0.9700
C16—H16B	0.9600	Cl1'—C39'	1.778 (10)
C16—H16C	0.9600	C39'—Cl2	1.770 (10)
C17—C22	1.392 (5)	C39'—H39C	0.9700
C17—C18	1.418 (5)	C39'—H39D	0.9700
C18—C19	1.360 (5)		
			
C20—S1—C23	104.20 (19)	C17—C18—H18	119.3
C28—S2—C31	103.65 (18)	C18—C19—C20	122.3 (4)
C6—N1—C7	108.5 (3)	C18—C19—H19	118.9
C6—N1—C32	110.6 (3)	C20—C19—H19	118.9
C7—N1—C32	110.7 (3)	C19—C20—C21	117.2 (4)
C15—N2—C14	110.8 (3)	C19—C20—S1	118.0 (3)
C16—N3—C1	114.2 (3)	C21—C20—S1	124.8 (3)
C16—N3—C8	115.9 (3)	C22—C21—C20	120.5 (4)
C1—N3—C8	105.3 (3)	C22—C21—H21	119.8
N3—C1—C2	102.3 (3)	C20—C21—H21	119.8
N3—C1—H1A	111.3	C17—C22—C21	122.0 (4)
C2—C1—H1A	111.3	C17—C22—H22	119.0
N3—C1—H1B	111.3	C21—C22—H22	119.0
C2—C1—H1B	111.3	S1—C23—H23A	109.5
H1A—C1—H1B	109.2	S1—C23—H23B	109.5
C17—C2—C1	116.8 (3)	H23A—C23—H23B	109.5
C17—C2—C3	115.9 (3)	S1—C23—H23C	109.5
C1—C2—C3	103.1 (3)	H23A—C23—H23C	109.5
C17—C2—H2	106.8	H23B—C23—H23C	109.5
C1—C2—H2	106.8	C5—C24—C25	131.1 (4)
C3—C2—H2	106.8	C5—C24—H24	114.4
C4—C3—C7	106.2 (3)	C25—C24—H24	114.4
C4—C3—C2	111.0 (3)	C26—C25—C30	115.5 (3)
C7—C3—C2	113.3 (3)	C26—C25—C24	119.6 (3)
C4—C3—C8	109.8 (3)	C30—C25—C24	124.9 (4)
C7—C3—C8	112.2 (3)	C27—C26—C25	123.2 (4)
C2—C3—C8	104.4 (3)	C27—C26—H26	118.4
O1—C4—C5	121.6 (3)	C25—C26—H26	118.4
O1—C4—C3	120.5 (3)	C26—C27—C28	121.1 (4)
C5—C4—C3	117.9 (3)	C26—C27—H27	119.5
C24—C5—C4	117.3 (4)	C28—C27—H27	119.5
C24—C5—C6	122.3 (3)	C27—C28—C29	116.6 (4)
C4—C5—C6	120.3 (3)	C27—C28—S2	125.6 (3)
N1—C6—C5	111.7 (3)	C29—C28—S2	117.8 (3)
N1—C6—H6A	109.3	C30—C29—C28	122.7 (4)
C5—C6—H6A	109.3	C30—C29—H29	118.6
N1—C6—H6B	109.3	C28—C29—H29	118.6
C5—C6—H6B	109.3	C29—C30—C25	120.9 (4)
H6A—C6—H6B	107.9	C29—C30—H30	119.6
N1—C7—C3	108.1 (3)	C25—C30—H30	119.6
N1—C7—H7A	110.1	S2—C31—H31A	109.5
C3—C7—H7A	110.1	S2—C31—H31B	109.5
N1—C7—H7B	110.1	H31A—C31—H31B	109.5
C3—C7—H7B	110.1	S2—C31—H31C	109.5
H7A—C7—H7B	108.4	H31A—C31—H31C	109.5
N3—C8—C9	112.0 (3)	H31B—C31—H31C	109.5
N3—C8—C15	110.0 (3)	N1—C32—C33	114.1 (3)
C9—C8—C15	100.7 (3)	N1—C32—H32A	108.7
N3—C8—C3	102.3 (3)	C33—C32—H32A	108.7
C9—C8—C3	119.1 (3)	N1—C32—H32B	108.7
C15—C8—C3	112.8 (3)	C33—C32—H32B	108.7
C10—C9—C14	117.5 (3)	H32A—C32—H32B	107.6
C10—C9—C8	132.2 (3)	C34—C33—C38	117.9 (3)
C14—C9—C8	110.1 (3)	C34—C33—C32	119.0 (3)
C11—C10—C9	120.2 (4)	C38—C33—C32	122.9 (4)
C11—C10—H10	119.9	C33—C34—C35	120.6 (4)
C9—C10—H10	119.9	C33—C34—H34	119.7
C10—C11—C12	121.3 (4)	C35—C34—H34	119.7
C10—C11—H11	119.4	C36—C35—C34	120.3 (4)
C12—C11—H11	119.4	C36—C35—H35	119.8
C13—C12—C11	119.6 (4)	C34—C35—H35	119.8
C13—C12—H12	120.2	C35—C36—C37	119.9 (4)
C11—C12—H12	120.2	C35—C36—H36	120.0
C14—C13—C12	117.8 (4)	C37—C36—H36	120.0
C14—C13—H13	121.1	C36—C37—C38	119.9 (4)
C12—C13—H13	121.1	C36—C37—H37	120.1
C13—C14—C9	123.6 (4)	C38—C37—H37	120.1
C13—C14—N2	126.4 (3)	C37—C38—C33	121.3 (4)
C9—C14—N2	110.0 (3)	C37—C38—H38	119.4
O2—C15—N2	124.4 (4)	C33—C38—H38	119.4
O2—C15—C8	127.3 (3)	Cl2—C39—Cl1	112.7 (3)
N2—C15—C8	108.2 (3)	Cl2—C39—H39A	109.0
N3—C16—H16A	109.5	Cl1—C39—H39A	109.0
N3—C16—H16B	109.5	Cl2—C39—H39B	109.0
H16A—C16—H16B	109.5	Cl1—C39—H39B	109.0
N3—C16—H16C	109.5	H39A—C39—H39B	107.8
H16A—C16—H16C	109.5	Cl2—C39'—Cl1'	110.8 (8)
H16B—C16—H16C	109.5	Cl2—C39'—H39C	109.5
C22—C17—C18	116.5 (3)	Cl1'—C39'—H39C	109.5
C22—C17—C2	119.2 (3)	Cl2—C39'—H39D	109.5
C18—C17—C2	124.3 (4)	Cl1'—C39'—H39D	109.5
C19—C18—C17	121.5 (4)	H39C—C39'—H39D	108.1
C19—C18—H18	119.3	C39—Cl2—C39'	19.7 (8)
			
C16—N3—C1—C2	177.5 (3)	C10—C9—C14—N2	−177.0 (3)
C8—N3—C1—C2	49.3 (3)	C8—C9—C14—N2	−0.4 (4)
N3—C1—C2—C17	−166.9 (3)	C15—N2—C14—C13	178.1 (3)
N3—C1—C2—C3	−38.6 (3)	C15—N2—C14—C9	−2.8 (4)
C17—C2—C3—C4	−97.5 (4)	C14—N2—C15—O2	−179.9 (3)
C1—C2—C3—C4	133.6 (3)	C14—N2—C15—C8	4.6 (4)
C17—C2—C3—C7	21.8 (5)	N3—C8—C15—O2	−61.5 (5)
C1—C2—C3—C7	−107.1 (3)	C9—C8—C15—O2	−179.9 (4)
C17—C2—C3—C8	144.2 (3)	C3—C8—C15—O2	52.0 (5)
C1—C2—C3—C8	15.3 (4)	N3—C8—C15—N2	113.8 (3)
C7—C3—C4—O1	−148.3 (3)	C9—C8—C15—N2	−4.5 (4)
C2—C3—C4—O1	−24.8 (5)	C3—C8—C15—N2	−132.6 (3)
C8—C3—C4—O1	90.1 (4)	C1—C2—C17—C22	−158.7 (3)
C7—C3—C4—C5	34.9 (4)	C3—C2—C17—C22	79.4 (4)
C2—C3—C4—C5	158.4 (3)	C1—C2—C17—C18	18.3 (5)
C8—C3—C4—C5	−86.7 (4)	C3—C2—C17—C18	−103.5 (4)
O1—C4—C5—C24	−16.0 (5)	C22—C17—C18—C19	−1.8 (5)
C3—C4—C5—C24	160.8 (3)	C2—C17—C18—C19	−178.9 (3)
O1—C4—C5—C6	166.1 (3)	C17—C18—C19—C20	1.2 (6)
C3—C4—C5—C6	−17.1 (5)	C18—C19—C20—C21	0.0 (6)
C7—N1—C6—C5	−52.7 (4)	C18—C19—C20—S1	178.9 (3)
C32—N1—C6—C5	−174.3 (3)	C23—S1—C20—C19	−176.4 (3)
C24—C5—C6—N1	−153.3 (3)	C23—S1—C20—C21	2.4 (4)
C4—C5—C6—N1	24.5 (5)	C19—C20—C21—C22	−0.5 (6)
C6—N1—C7—C3	76.1 (3)	S1—C20—C21—C22	−179.3 (3)
C32—N1—C7—C3	−162.3 (3)	C18—C17—C22—C21	1.2 (5)
C4—C3—C7—N1	−64.0 (4)	C2—C17—C22—C21	178.5 (3)
C2—C3—C7—N1	173.9 (3)	C20—C21—C22—C17	−0.1 (6)
C8—C3—C7—N1	56.0 (4)	C4—C5—C24—C25	−179.2 (3)
C16—N3—C8—C9	65.9 (4)	C6—C5—C24—C25	−1.4 (6)
C1—N3—C8—C9	−166.9 (3)	C5—C24—C25—C26	154.6 (4)
C16—N3—C8—C15	−45.2 (4)	C5—C24—C25—C30	−26.8 (7)
C1—N3—C8—C15	82.0 (3)	C30—C25—C26—C27	0.7 (6)
C16—N3—C8—C3	−165.3 (3)	C24—C25—C26—C27	179.4 (4)
C1—N3—C8—C3	−38.1 (3)	C25—C26—C27—C28	−2.0 (6)
C4—C3—C8—N3	−106.3 (3)	C26—C27—C28—C29	2.5 (6)
C7—C3—C8—N3	135.8 (3)	C26—C27—C28—S2	−177.4 (3)
C2—C3—C8—N3	12.8 (3)	C31—S2—C28—C27	−3.5 (4)
C4—C3—C8—C9	17.9 (4)	C31—S2—C28—C29	176.5 (3)
C7—C3—C8—C9	−100.0 (4)	C27—C28—C29—C30	−1.9 (6)
C2—C3—C8—C9	136.9 (3)	S2—C28—C29—C30	178.1 (3)
C4—C3—C8—C15	135.6 (3)	C28—C29—C30—C25	0.6 (6)
C7—C3—C8—C15	17.7 (4)	C26—C25—C30—C29	0.0 (6)
C2—C3—C8—C15	−105.3 (3)	C24—C25—C30—C29	−178.6 (4)
N3—C8—C9—C10	61.9 (5)	C6—N1—C32—C33	−70.4 (4)
C15—C8—C9—C10	178.8 (4)	C7—N1—C32—C33	169.3 (3)
C3—C8—C9—C10	−57.4 (5)	N1—C32—C33—C34	157.8 (3)
N3—C8—C9—C14	−114.0 (3)	N1—C32—C33—C38	−26.7 (5)
C15—C8—C9—C14	2.9 (4)	C38—C33—C34—C35	−2.0 (6)
C3—C8—C9—C14	126.7 (3)	C32—C33—C34—C35	173.7 (4)
C14—C9—C10—C11	−1.5 (5)	C33—C34—C35—C36	0.3 (6)
C8—C9—C10—C11	−177.2 (4)	C34—C35—C36—C37	1.4 (6)
C9—C10—C11—C12	−0.6 (6)	C35—C36—C37—C38	−1.3 (6)
C10—C11—C12—C13	2.3 (6)	C36—C37—C38—C33	−0.4 (6)
C11—C12—C13—C14	−1.7 (5)	C34—C33—C38—C37	2.1 (6)
C12—C13—C14—C9	−0.5 (6)	C32—C33—C38—C37	−173.4 (4)
C12—C13—C14—N2	178.4 (3)	Cl1—C39—Cl2—C39'	61 (2)
C10—C9—C14—C13	2.1 (5)	Cl1'—C39'—Cl2—C39	−58.0 (14)
C8—C9—C14—C13	178.7 (3)		

### Hydrogen-bond geometry (Å, °)

**Table:**

*D*—H···*A*	*D*—H	H···*A*	*D*···*A*	*D*—H···*A*
C1—H1A···O2	0.97	2.53	3.149 (5)	122
C7—H7B···O2	0.97	2.37	2.971 (5)	119
C10—H10···O1	0.93	2.57	3.220 (5)	127
C39—H39A···N3	0.97	2.44	3.405 (10)	175
C39—H39B···O1	0.97	2.53	3.140 (7)	121
